# Efficacy and safety of external treatment of traditional Chinese medicine combined with conventional treatment for idiopathic pulmonary fibrosis: A systematic review and meta-analysis

**DOI:** 10.1097/MD.0000000000045018

**Published:** 2025-10-10

**Authors:** Yalan Li, Xiangyi Wu, Yan Xue, Wei Zhang

**Affiliations:** a Department of Pulmonary Diseases, ShuGuang Hospital Affiliated to Shanghai University of Traditional Chinese Medicine, Shanghai, China; b Shanghai University of Traditional Chinese Medicine, Shanghai, China.

**Keywords:** clinical efficacy, external treatment, idiopathic pulmonary fibrosis, meta-analysis, systematic review, traditional Chinese medicine

## Abstract

**Background:**

Idiopathic pulmonary fibrosis (IPF) with high mortality posed a great threat to public health, so seeking for alternative therapies for IPF is a growing concern. In China, external treatment of traditional Chinese medicine (TCM), an effective supplementary intervention has shown potential value. This study aims to evaluate the efficacy and safety of external treatment of TCM combined with conventional treatment for IPF.

**Methods:**

Eight databases were searched from their inception to March 2024, to collect randomized controlled trials, of external treatment of TCM combined with conventional treatment for IPF. Two researchers independently conducted study selection and data extraction. The quality of the study was evaluated using the Cochrane Risk of Bias Tool, and data were analyzed using RevMan 5.4 software.

**Results:**

The meta-analysis included 11 studies with a total of 981 participants with IPF. The results of it showed that forced vital capacity, diffusion capacity for carbon monoxide, 6-minute walk distance and arterial partial pressure of oxygen were significantly improved in the group of external treatment combined with conventional treatment (experimental group) than the group receiving conventional treatment only (control group). Symptom score of St George’s Respiratory Questionnaire and activity score of St George’s Respiratory Questionnaire were significantly decreased in the experimental group than the control group.

**Conclusion:**

External treatment, as an effective supplementary intervention, combined with conventional treatment shows promising efficacy and safety in treating IPF. However, the limited number and quality of the included studies caused the limitations of these results. More high-quality randomized controlled trials are required to confirm the conclusion in the future.

## 1. Introduction

Idiopathic pulmonary fibrosis (IPF) is an irreversible interstitial lung disease characterized by progressive dyspnea and decreasing exercise capacity.^[1]^ Its mortality is high and its survival time is short. Once diagnosed, the median survival time of patients is only 3 to 5 years.^[2]^ Pirfenidone and nintedanib, recommended by clinical practice guidelines, slow down disease progression. However, patients often reduce dosage or stop treatment because of side effects and high cost, which makes the therapeutic effect not meet expectations.^[1,3–5]^ Therefore, more treatments need to be further explored.

Traditional Chinese medicine (TCM) has thousands of years of experience in treating IPF, by alleviating symptoms and slowing down the decline in lung function in patients with IPF.^[6,7]^ TCM treatment consists of 2 systems: internal treatment (oral route of administration) and external treatment, which have the ability to tonify Qi and nourish Yin, promote blood circulation, resolve stasis, and dredge collaterals, and the mechanism of treating IPF involves multi-target and multichannel comprehensive regulation, which mainly include anti-inflammatory, anti-oxidation, anti-fibrosis and immunomodulation.^[8]^ Clinical trials on the intervention of IPF by external treatment combined with conventional treatment are gradually increasing, but there is no systematic evaluation report on the clinical efficacy of it.^[9]^ Therefore, this meta-analysis aims to systematically assess the efficacy and safety of external treatment combined with conventional treatment for IPF.

## 2. Methods

This systematic review and meta-analysis have been registered on the International Prospective Register of Systematic Reviews (PROSPERO) on January 25, 2024 (registration number CRD42024502218).

### 2.1. Literature search strategy

We searched CNKI, Wanfang database, China Biomedical Literature Database and VIP Information Resource System, Cochrane Library, Embase, Pubmed, Web of science for articles published from inception to March, 2024. The search terms included idiopathic pulmonary fibrosis, pulmonary fibrosis, acupuncture, electroacupuncture, moxibustion, iontophoresis, acupoint application, acupoint sticking, acupoint injection, Cupping, Tuina, Baduanjin, Taiji, Taichi, Qigong, and Daoyin. We used medical subject heading terms and free keywords to identify potential publications.

### 2.2. Inclusion criteria

Study design: all randomized controlled trials were included, with language restricted to Chinese or English. Population: patients with IPF, diagnosed by ATS/ERS/JRS/ALAT IPF guidelines. Intervention and comparison: the control group received conventional treatment (such as Western medicine or oral Chinese medicine), while the experimental group received external treatment combined with conventional treatment. Outcome: forced vital capacity (FVC); diffusion capacity for carbon monoxide (DL_CO_); 6-minute walk distance (6MWD); St George’s Respiratory Questionnaire (SGRQ) scores (symptom, activity and impact); and arterial partial pressure of oxygen (PaO_2_).

### 2.3. Exclusion criteria

Non-RCT study; trial data were incomplete; interventions did not meet the inclusion criteria; and key efficacy indicators were missing.

### 2.4. Literature screening and data extraction

Literature screening was conducted independently by 2 researchers. Discrepancies were resolved through negotiation or consultation with a third party. The process included: Import retrieved literature into EndNote X9 to remove duplicates. Primary screen based on titles and abstracts to exclude irrelevant studies. Secondary screen involved full-text review to further exclude studies not meeting inclusion criteria. Check the studies finally included and extract data.

Two researchers independently extracted information using a standardized form included: first author’s name, publication year, gender, sample size, intervention, and outcome (mean and standard deviation).

### 2.5. Evaluation of literature quality

RCTs included in the study were assessed for risk of bias using the risk of bias assessment tool provided by the Cochrane Handbook for Systematic Evaluators, which evaluates: randomization; allocation concealment; blinding of participants and assessors; completeness of outcome data; selective reporting of results; other potential biases. Assessment results were categorized as “low risk,” “unclear,” or “high risk.”

### 2.6. Statistical analysis

Meta-analysis was performed using RevMan 5.4 software (Cochrane Collaboration, London, United Kingdom), and the difference was considered statistically significant at *P* < .05. Key aspects included: Measurement information used weighted mean difference (MD) or standardized mean difference (SMD) for differing units. Effect sizes were reported with 95% confidence intervals (CI), and forest plots were generated. Heterogeneity was assessed using the *I*^2^ test. A fixed-effects model was applied if *I*^2^ ≤ 50% and/or *P* ≥ .1 in the *Q* test, indicating low heterogeneity. If *I*^2^ > 50% and/or *P* < .1 in the *Q* test, it was suggested that the heterogeneity was high, and a random-effects model was selected and further processed by subgroup analysis or sensitivity analysis.

### 2.7. Ethical review

This study was approved by the ethics committee of Shuguang Hospital affiliated to Shanghai University of TCM, Shanghai, China. All methods were carried out in accordance with the Declaration of Helsinki. As the meta-analysis study was based on published literature data, and did not involve individual patient data. Therefore, informed consents of the patients were not necessary.

## 3. Results

### 3.1. Study selection

**Figure 1.**
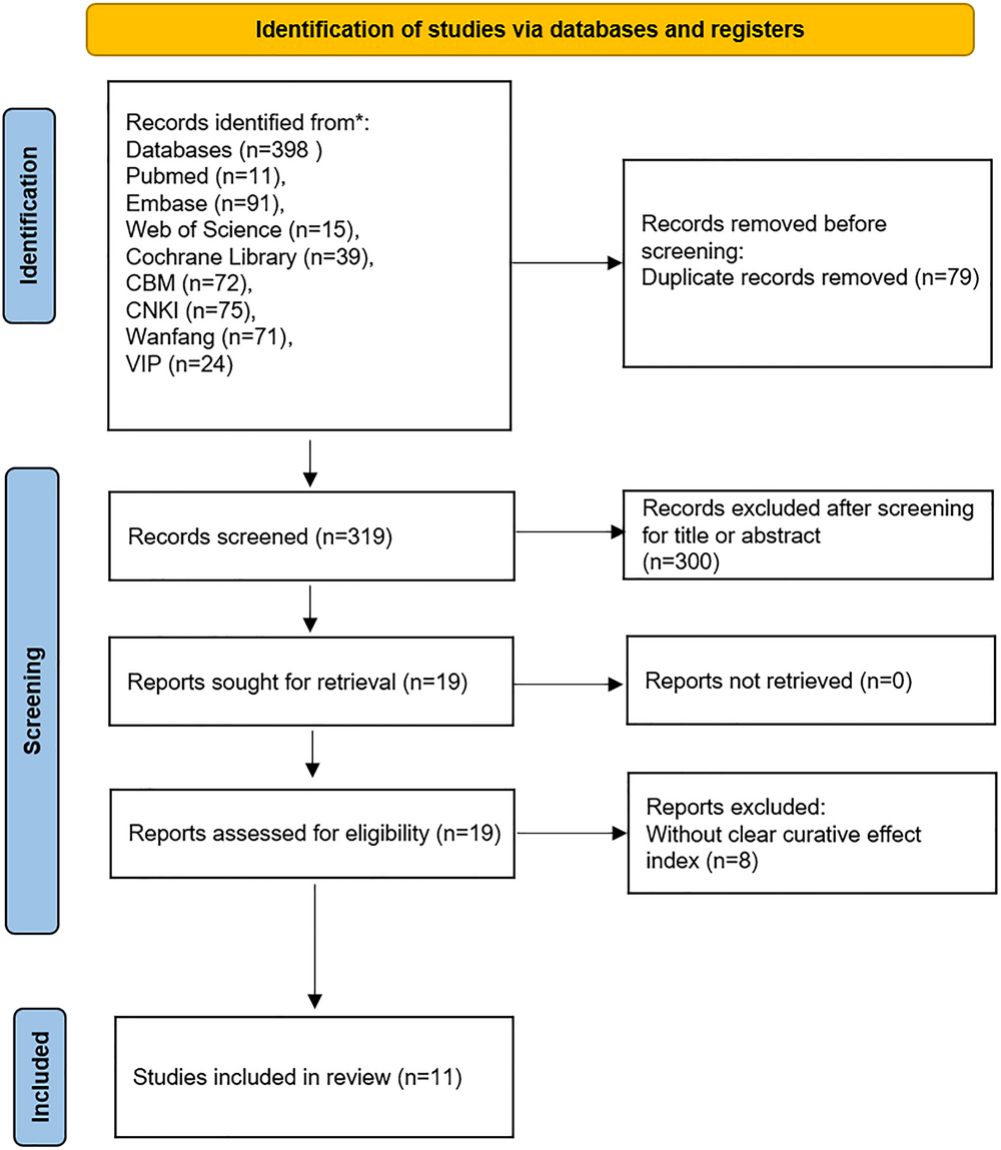
Flowchart of the study screening process.

A total of 398 studies were identified through the search strategy. Initially, 79 duplicate studies were removed. After reviewing the titles and abstracts, 300 studies unrelated to clinical researches on IPF were excluded. An additional 8 studies lacking clear efficacy indicators were also excluded. Ultimately, 11 studies were included in this meta-analysis. The specific screening process is shown in Figure 1.

### 3.2. Characteristics of the included studies

The 11 included studies^[10–20]^ comprised 981 participants (489 in the experimental group and 492 in the control group). All studies were conducted in China, with 1 study published in English and the remaining ten in Chinese. The external treatment included herbal iontophoresis, acupoint application, acupoint injection, acupuncture, and Qigong. Eight studies reported FVC, 8 studies reported DL_CO_, 4 studies reported 6MWD, 2 studies reported SGRQ, and 6 studies reported PaO_2_. All indicators were measured after the intervention. Detailed information is provided in Table 1.

**Table 1:** Characteristics of included studies.

Author, year	Sample number (e.g./CG)	Gender (M/F)	Age (e.g./CG)	Intervention methods (e.g./CG)	Treatment duration	Outcome
Tian, 2019	112 (56/56)	48/64	45.33 ± 12.37/45.62 ± 12.10	Acupoint application combined with conventional treatment/conventional treatment	3 mo	①, ②, ⑤
Song, 2022	60 (30/30)	27/33	53.19 ± 8.62/52.37 ± 8.28	TCM iontophoresis combined with conventional treatment/conventional treatment	4 wk	①, ②, ④
Zhang, 2011	86 (43/43)	49/37	59 ± 8/58 ± 8	TCM iontophoresis combined with conventional treatment/conventional treatment	3 mo	②
Shen, 2022	124 (62/62)	73/51	64.5 ± 4.64/65.5 ± 4.66	Acupoint injection combined with conventional treatment/conventional treatment	3 mo	①, ②, ④
Zhou, 2021	77 (39/38)	44/33	58 ± 7/58 ± 5	Heat-sensitive moxibustion combined with conventional treatment/conventional treatment	3 mo	①, ②, ④
Ji, 2015	32 (16/16)	25/7	63.41 ± 3.24/66.41 ± 3.55	TCM iontophoresis combined with conventional treatment/conventional treatment	3 mo	②, ③, ④
Xu, 2010	87 (42/45)	45/42	56 ± 11/55 ± 11	Acupuncture and moxibustion combined with conventional treatment/conventional treatment	3 mo	①, ④
Liu, 2021	64 (32/32)	34/30	56.02 ± 8.37/57.28 ± 8.48	Indirect moxibustion with ginger combined with conventional treatment/conventional treatment	3 mo	①, ③, ④
Huang, 2021	158 (79/79)	97/61	62.35 ± 7.55/61.98 ± 7.96	Thunder-fire moxibustion combined with conventional treatment/conventional treatment	35 d	①, ②
Miao, 2021	63 (32/31)	NA	66 ± 11/67 ± 10	Daoyin/conventional treatment	2 mo	①, ②, ③, ⑤
Li, 2019	116 (58/58)	72/44	61 ± 8/64 ± 6	Umbilical cord moxibustion combined with conventional treatment/conventional treatment	3 mo	③

CG = control group, EG = experimental group, F = female, M = male.

①: FVC = forced vital capacity, ②: DL_CO_ = diffusion capacity for carbon monoxide, ③: 6MWD = 6-minute walk distance, ④: PaO_2_ = arterial partial pressure of oxygen, ⑤: SGRQ = St George’s Respiratory Questionnaire.

### 3.3. Quality assessment

**Figure 2.**
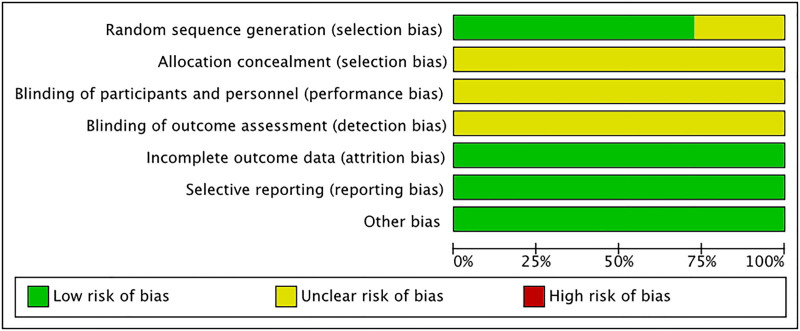
Risk of bias graph.

**Figure 3.**
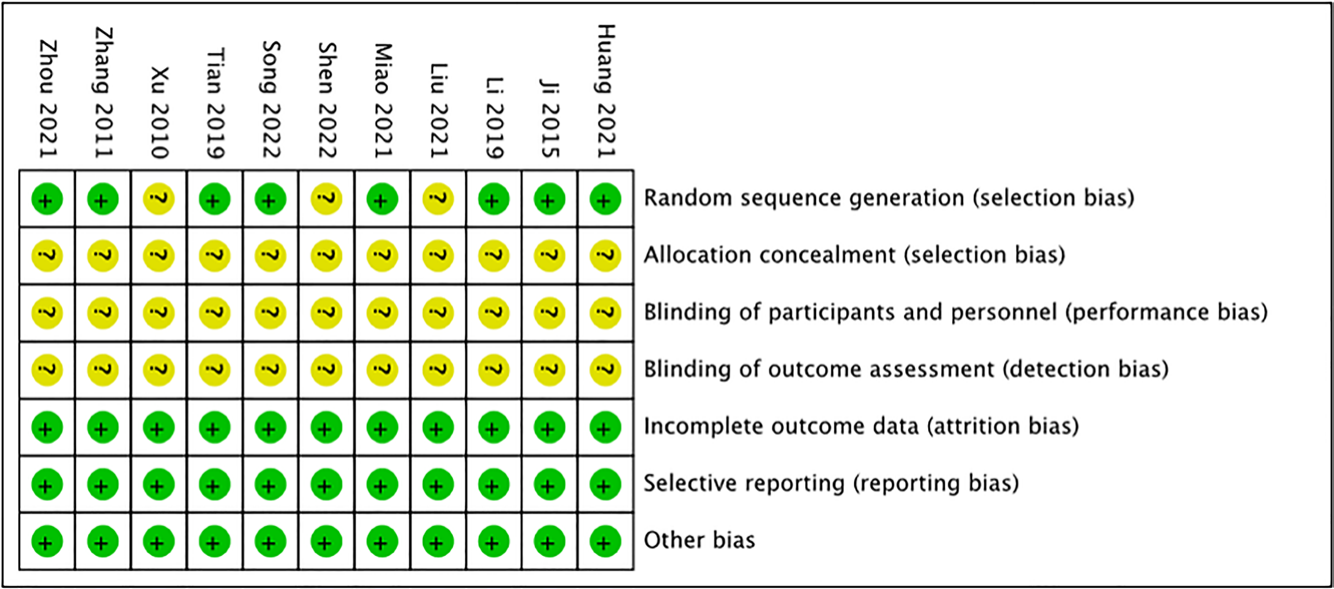
Risk of bias summary.

We assessed the quality of the included studies using the Cochrane Risk of Bias tool. Among the 11 included RCTs, 8 studies reported specific randomization schemes and were rated as “low risk.” while 3 studies did not specify the randomization method and were rated as “unclear.” None of the studies reported allocation concealment, blinding of participants and personnel, or blinding of outcome assessment, thus these domains were rated as “unclear.” No significant bias was found in the other aspects. Figures 2 and 3 present the risk of bias assessment for each study.

### 3.4. Results of meta-analysis

#### 3.4.1. Lung function

**Figure 4.**
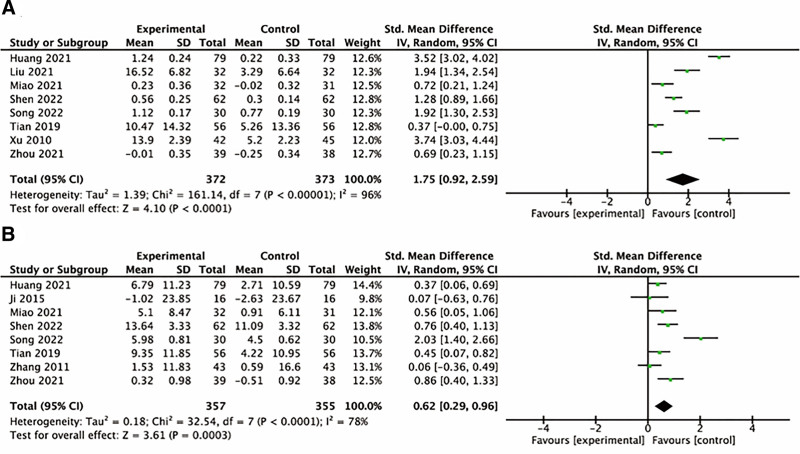
Forest plot of comparison: pulmonary function tests. (A) FVC change. (B) DL_CO_ change. DLCO = diffusion capacity for carbon monoxide, FVC = forced vital capacity.

Eight studies compared FVC and DL_CO_ of patients in experimental group and control group. The meta-analysis revealed heterogeneity among the studies (*I*^2^ = 96%; *I*^2^ = 78%). Sensitivity analysis indicated that removing any single study did not eliminate heterogeneity. There is only 1 study in some subgroups, so it is not suitable for subgroup analysis. A random effect model is used to analyze the combined effect. The result showed external treatment of TCM as an effective supplementary intervention slowed the decline in FVC of patients with IPF (SMD = 1.75, 95% CI [0.92–2.59], *P* < .0001), and improved DL_CO_ (SMD = 0.62, 95% CI [0.29–0.96], *P* = .0003) (Fig. 4).

#### 3.4.2. Exercise capacity

**Figure 5.**
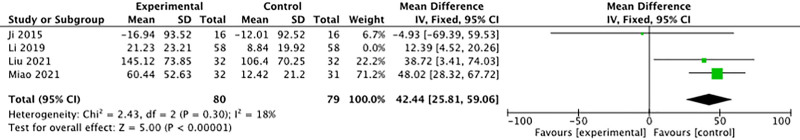
Forest plot of comparison: 6MWD change. 6MWD = 6-minute walk distance.

Four studies reported the results of 6MWD. Heterogeneity test revealed *I*^2^ = 76%, *P* = .005, indicating substantial heterogeneity. Sensitivity analysis suggested that the heterogeneity might be attributed to the study by Li Bin.^[20]^ After excluding this study, heterogeneity test on the remaining 3 studies^[15,17,19]^ showed *I*^2^ = 18%, *P* = .3, indicating low heterogeneity. Therefore, a fixed-effect model was used for analysis. The results showed that external treatment significantly improved the 6MWD in patients (MD = 42.44, 95% CI [25.81, 59.06], *P* < .00001) (Fig. 5).

#### 3.4.3. Quality of life

**Figure 6.**
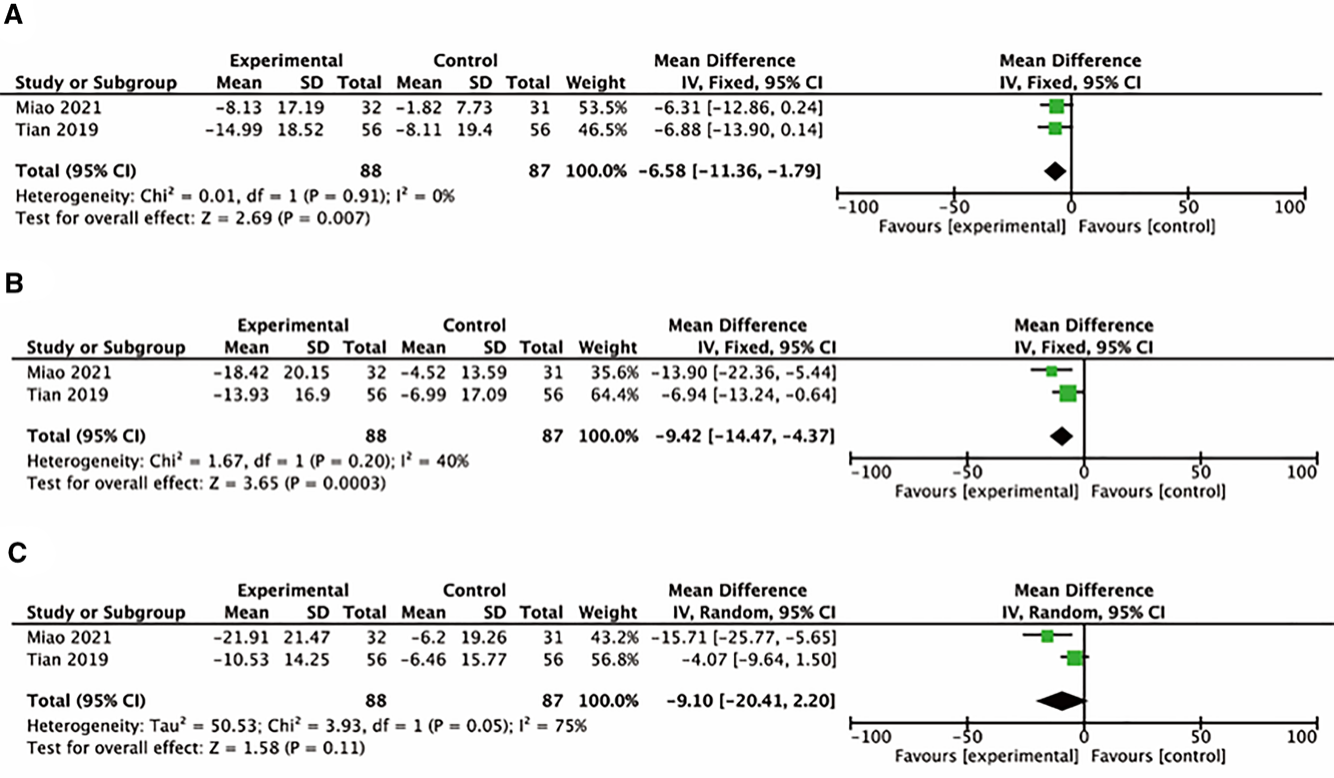
Forest plot of comparison: SGRQ score. (A) Activity score change of SGRQ. (B) Symptoms score change of SGRQ. (C) Impact score change of SGRQ. SGRQ = St George’s Respiratory Questionnaire.

The meta-analysis of quality of life score revealed that compared with the control group, external treatment significantly reduced symptom and activity score of SGRQ (lower SGRQ scores indicate better quality of life), with statistically significant differences (*P* < .05). The 2 studies had low heterozygosity (heterozygosity test, chi^2^ = 0.01, *P* = .91, *I*^2^ = 0%; chi^2^ = 1.67, *P* = .2, *I*^2^ = 40%). When the fixed-effect model was used to merge MD values, the pooled MD were −6.58 (95% CI [‐11.36, −1.79], *Z* = 2.69, *P* = .007); −9.42 (95% CI [‐14.47, −4.37], *Z* = 3.65, *P* = .0003). However, no statistically significant difference was found in impact change score of SGRQ (*P* > .05). The study had high heterozygosity (heterozygosity test, chi^2^ = 3.93, *P* = .05, *I*^2^ = 75%). When the random effect model was used to merge MD values, the pooled MD were −9.10 [95% CI (−20.41, 2.20), *Z* = 1.58, *P* = .11] (Fig. 6).

#### 3.4.4. Other results

**Figure 7.**
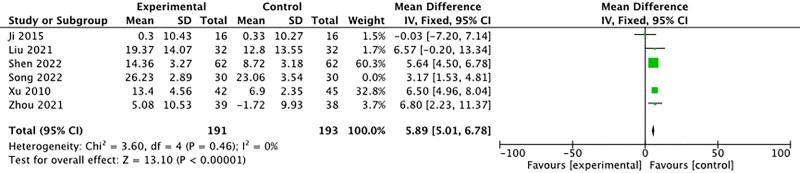
Forest plot of comparison: PaO_2_ change. PaO2 = arterial partial pressure of oxygen.

Six studies reported the results of PaO_2_ at rest. The meta-analysis indicated heterogeneity among the studies (*I*^2^ = 58%, *P* = .04). Sensitivity analysis using Revman software suggested that the heterogeneity might be attributed to the study by Song Dongsheng.^[11]^ After excluding this study, the remaining studies exhibited no significant heterogeneity (*I*^2^ = 0%, *P* = .46), allowing for analysis using a fixed-effect model. The results demonstrated that external treatment significantly improved PaO_2_ in patients with IPF compared to the control group (MD = 5.89, 95% CI [5.01, 6.78], *P* < .00001) (Fig. 7).

#### 3.4.5. Safety

Six studies^[10,12–15,19]^ reported safety evaluation. Four studies^[10,12,14,15]^ reported that there were no adverse reactions in the experimental group and the control group. One study^[13]^ reported that patients in the experimental group had mild dry mouth and drowsiness. One study^[19]^ reported that 1 patient in the experimental group had acute exacerbation events, and 9 patients in the control group had acute exacerbation events. The other 5 studies^[11,16–18,20]^ did not include any safety evaluation.

### 3.5. Publication bias

Publication bias was not assessed for outcomes of the experimental group and the control group because the included studies were <10 for both comparisons.

## 4. Discussion

IPF is a fatal lung disease with unknown etiology and complicated pathogenesis. Lung transplantation may be the only option to cure IPF. However, this method cannot be widely used in clinical practice due to the limitation of donor organs and surgical risks.^[1]^ Therefore, the current goal of treating IPF is to slow down the disease progression, improve the quality of life of patients, and prolong the survival time of patients. The typical changes of lung function in patients with IPF are restrictive ventilation disorder and diffusion impairment, and the main manifestations are the decline of FVC and DL_CO_.^[21,22]^ With the progress of the disease, cardiopulmonary function and exercise capacity are getting worse and worse, which has a great impact on patients’ life. 6MWD objectively evaluates the functional motor abilities of patients with lung diseases, and we can predict the survival rate of patients with IPF through its changes.^[23,24]^ And the SGRQ questionnaire evaluates the impact of respiratory problems of chronic airflow limitation on patients’ quality of life in 3 dimensions: symptom, activity and impact.^[25]^ In addition, patients with IPF may develop resting hypoxemia or even respiratory failure in the later stages of the disease due to the long-term damage of lung tissues, which leads to a decrease in gas exchange capacity. Therefore, we chose the changes in FVC, DL_CO_, 6MWD, SGRQ scores and PaO_2_ before and after treatment as the assessment indexes in this study.

External treatment of TCM is a clinical intervention method that embodies the characteristics of TCM. It has the advantages of diverse treatment modalities, safety, effectiveness and easy operation. It usually is used to treat IPF, combination with conventional treatment. Eleven randomized controlled trials were included in this study, including herbal iontophoresis, acupoint application, acupoint injection, acupuncture and Daoyin. Iontophoresis of TCM is to deliver directly TCM to local tissues or blood through the skin, so as to maximize the drug concentration and reduce the adverse stimulation of drugs to the gastrointestinal tract.^[26]^ Acupoint sticking is the combination of TCM with specific acupoints to achieve the purpose of dual stimulation of drugs and acupoints, which is often used in the treatment of some chronic diseases.^[27]^ Similarly, because the drug is absorbed through the skin, some adverse reactions caused by the stimulation of the drug to the gastrointestinal tract are greatly avoided. In addition, acupoint injection therapy is a kind of TCM treatment similar to acupoint sticking. Different from the above therapies, acupuncture and Daoyin are 2 nondrug therapies. Acupuncture is a TCM therapy widely used in the world, while Daoyin, as a TCM lung rehabilitation method, has a relatively limited range of application, which mainly regulates the whole body qi through specific actions to achieve the purpose of curing diseases, and has been included in IPF TCM rehabilitation guidelines at present.^[28]^ Previous studies have shown that Daoyin aims to improve respiratory function, increase exercise tolerance, and reduce the frequency of acute exacerbations, and the related clinical advantages have been confirmed by many studies.^[19,29,30]^

The results of this meta-analysis showed the external treatment combined with conventional treatment was superior to conventional treatment only in improving FVC, DL_CO_, 6MWD, SGRQ score and PaO_2_ index, so the external treatment had positive effects on improving patients’ lung function, exercise capacity and quality of life in a way.

This meta-analysis also had some limitations: firstly, there was only small number of available studies and small sample sizes, so that publication bias could not be ruled out; secondly, there was heterogeneity in some of these outcomes to a certain extent, which may be related to the measurements of the outcomes, and the methods of intervention. For example, in some studies,^[10–14,18,19]^ the lung function was expressed in terms of FVC or DL_CO_, whereas in other studies,^[13,15,19,30]^ it was expressed in terms of FVC% pred or DL_CO_% pred; furthermore, all studies were conducted in China, so the results might not be applicable to other countries or ethnicities. Finally, we did not perform subgroup analyses and funnel plot analyses due to limitations in the number of included studies and the type of specific treatment measures.

Therefore, more high-quality clinical studies with rigorous design and consistent assessment of indicators are still needed in the future to further explore the efficacy and safety of external treatment combined with conventional treatment for IPF.

## 5. Conclusion

In conclusion, this meta-analysis suggested that external treatment could effectively improve lung function, exercise capacity and quality of life in patients with IPF, showing good prospects for application; however, the quality of the existing studies is poor, and higher-quality clinical studies are still needed to verify them in the future, in order to confirm the efficacy of external treatment interventions in patients with IPF.

## Author contributions

**Formal analysis:** Xiangyi Wu.

**Funding acquisition:** Wei Zhang.

**Methodology:** Yalan Li.

**Software:** Yalan Li, Xiangyi Wu, Yan Xue.

**Writing – original draft:** Yalan Li.

**Writing – review & editing:** Wei Zhang.
